# Comprehensive analysis and experiment validation of five cuproptosis-related genes in prognosis, immune infiltration and metabolic characterization of pancreatic cancer

**DOI:** 10.1371/journal.pone.0323458

**Published:** 2025-05-14

**Authors:** Qianxi Deng, Kun Yang, Qiaoling Liao, Xueli Tang, Honglin Quan, Guojun Yuan, Xia Hu, Zheng Jiang, Linju Wu

**Affiliations:** 1 Department of Gastroenterology, The Third Hospital of Mianyang (Sichuan Mental Health Center), Mianyang, Sichuan, China; 2 Department of Gastroenterology, The First Affiliated Hospital of Chongqing Medical University, Chongqing, China; 3 The Second Department of Severe Psychiatry, The Third Hospital of Mianyang (Sichuan Mental Health Center), Mianyang, Sichuan, China; 4 Department of Science and Technology, The Third Hospital of Mianyang (Sichuan Mental Health Center), Mianyang, Sichuan, China; 5 Department of Anesthesiology, The Third Hospital of Mianyang (Sichuan Mental Health Center), Mianyang, Sichuan, China; Xiangya Hospital Central South University, CHINA

## Abstract

**Background:**

Cuproposis is a new-found mechanism of cell death, and the role of cuproposis-related genes (CRGs) in pancreatic cancer prognosis remains uncertain.

**Methods:**

DECRGs were identified from TCGA and GTEx databases. Five OS-associated hub genes were screened using Cox regression and LASSO analyses. A prognostic model was constructed and validated by survival analysis. GSEA, gene mutation, small-molecule drugs, immune-infiltrating and TF/miRNA/mRNA network were investigated to determine the underlying mechanism of 5-CRGs. In addition, RT-qPCR, and WB were applied to validate the expression of 5-CRGs. CCK8, colony formation and transwell assays were used to prove the function of LIPT1 in PC.

**Results:**

PDP1, DLAT, DBT, LIAS, and LIPT1 were screened as hub genes. 5-CRGs prognostic model established the low-risk population has a longer OS. There was a high the risk score value for the prediction in clinicopathological features. The forest plots showed that age, N stage and the RiskScore were the significant independent risk indicators. T cells CD4 memory resting and Mast cells are the amplest immune cell subpopulations in the high-score individuals. The expression of 5 CRGs exhibited significant differences in PC cell lines and tissues, LIPT1-knockdowning inhibited proliferation and invasion of pancreatic cancer cell lines.

**Conclusion:**

Five CRGs relevant to pancreatic cancer prognosis were identified. Meanwhile, a new and accurate five CRGs prognostic model of pancreatic cancer was constructed. In addition, LIPT1 may promote proliferation, invasion and migration of pancreatic cancer cell lines. This may have a specific guiding value for future development of precise anti-cancer treatment strategies.

## 1. Introduction

Pancreatic cancer is a malignant tumor with unfavorable prognosis and high mortality and become the third dominating cause for tumor-associated mortality, with the lowest 5-year survival rate among all tumors of 12% [1]. In most cases, the low survival rate is partly ascribed to the delayed diagnosis, since only about 20% of cases can be determined with early-stage, surgically resectable tumor at diagnosis [2]. Hence, it is of significance to achieve the early identification of this malignancy to raise the chance of surgery and provide early treatment through developing strategies that enable early discovery and screening. Meanwhile, there is an urgency to identify the mechanisms of pancreatic carcinogenesis.

Copper, an essential element for human survival, participates in various biological processes such as cell growth and metabolism. Cells absorb, transmit, internalize, and store copper to achieve active copper homeostasis [3]. Copper is known as a cofactor essential for enzymes that modulate multiple key cellular functions, encompassing mitochondria respiratory function and antioxidant defense as well as the biosynthesis of substances such as hormones, neurotransmitters, and pigments, while oxidative stress and cytotoxicity will be triggered owing to the copper storage imbalance [4,5]. It has been reported that changes in copper levels can lead to cancer initiation and progression [4,6,7]. Recent studies have shown that copper overload leads to a new-found cell death program named cuproptosis. The specific mechanism is that copper directly binds to lipid acylated proteins in the tricarboxylic acid (TCA) cycle results in acute proteotoxic stress, resulting in mitochondrial metabolic abnormalities and eventually cell death [8,9]. Cuproptosis is strongly associated with cancer progression and is expected to be a novel therapeutic target with specificity against cancer cells [10,11]. Currently, how cuproptosis participates in the pathogenesis of pancreatic cancer or affects disease development or progression remains to be deeply explored. In light of this situation, comprehensively analyzing cuproptosis-related genes (CRGs) in pancreatic tumor tissues can help to identify diagnostic and therapeutic molecular targets for this malignancy.

In a previous study, the differential expression pattern of CRGs together with their copy number variation (CNV) in pancreatic cancer have been identified through bioinformatics strategies. Accordingly, a prognostic risk model dependent on five CRGs was developed by LASSO and multi-variable cox regression analyses. Pancreatic cancer samples were separated based on risk scores, and a significant prolonged overall survival (OS) of the low-risk population was detected than that of the high-risk population. Afterwards, the sensitivity and specificity of our model was calculated according to Kaplan–Meier and receptor operating characteristic (ROC) curves. Functional enrichment analysis and immune infiltration analysis were implemented to reveal potential correlations between CRGs and metabolic pathways and tumor immune microenvironment (TIME). The expression profiles of 5 CRGs at mRNA levels were verified by real time-PCR and LIPT1 at protein levels were proved via using WB. Finally, the function of LIPT1 in pancreatic cancer cell lines were validated by knocking down its expression level, which might promote proliferation, invasion and migration of pancreatic cancer. In conclusion, this study provides valuable insights into seeking new therapeutic interventions, as well as therapeutic biomarkers for this malignancy.

## 2. Methods

### 2.1. CRG screening and data acquisition

Sixteen cuproptosis-related genes (CRGs) were identified based on previous literature [9]. Transcriptomic data, clinical information, copy number variation (CNV), and single nucleotide variation (SNV) data for pancreatic cancer patients were obtained from the TCGA-PAAD project (The Cancer Genome Atlas Pancreatic Adenocarcinoma cohort, accessed via https://portal.gdc.cancer.gov/), while transcriptional profiles of normal pancreatic tissues were acquired from the GTEx database (Genotype-Tissue Expression Project, version v8, https://gtexportal.org/). Data were retrieved in May 2023 using TCGA database version 36.0. The TCGA-PAAD dataset initially included 178 pancreatic cancer patients. After excluding cases with incomplete transcriptomic or survival data, 162 tumor samples and 167 paracancerous tissues were retained for analysis. Clinical characteristics of the included samples were as follows: male (52.3%, 85/162), female (47.7%, 77/162); median age 65 years (range 35–88); TNM stage distribution: stage I (12.9%), stage II (63.6%), stage III/IV (23.5%). All data usage complied with the TCGA and GTEx Data Use Agreements.

### 2.2. Identification of OS-associated CRGs and decision curve analysis

DCA was used to evaluate the clinical net benefit of the model by comparing its predictive value against the “treat-all” or “treat-none” strategies across different threshold probabilities. Specifically, the net benefit was calculated as:


Net Benefit = (True Positives/N) − (False Positives/N) × (pt/(1−pt))

where N is the total sample size, and pt is the threshold probability (i.e., the minimum risk probability at which clinical intervention is warranted). A DCA curve closer to the top-left corner indicates higher clinical utility of the model within specific threshold ranges. High- and low- CRGs scoring groups were assigned relying on the risk scores CRGs, and then survival estimates of these two populations generated using R packages “survival”, “survminer” and “ggplot2” were compared using Kaplan–Meier curves [12]. Uni-variable Cox proportional risk regression was adopted to identify OS-associated CRGs, in TCGA-PAAD (n = 162), and genes showing a significance level of *p* < 0.05 were incorporated into the LASSO-Cox regression, yielding candidate genes after 10-fold cross-validation. Predictive ability of pancreatic cancer diagnosis rate and OS by time-dependent ROC curves was evaluated through utilizing R packages “timeROC” and “ggplot2”.

Decision curve analysis (DCA) is a method for evaluating substitutable diagnostic and prognostic strategies [13] and was generated using the R package “ggDCA” in this work.

### 2.3. Nomogram construction and calibration curve plotting

OS probabilities by uni- and multi-variable Cox regression were estimated using models constructed from CRGs as prognostic indicators in combination with other clinical factors, and conventional column plots with calibration curves wereconstructed based on these data. Final results were visualized using R packages “forestplot” and “rms”[14].

### 2.4. Functional enrichment analysis of hub genes and Heatmap and TF/mRNA/miRNA network map

Biological processes associated with CRGs in pancreatic cancer were identified and validated through Gene Ontology (GO) and Kyoto Encyclopedia of Genes and Genomes (KEGG). The enrichment was realized by R package’s “clusterProfiler”. Then, the enrichment graph and gene-concept network were generated through R package “enrichplot” (Bioconductor)[15].

In this study, the R software “pheatmap “ package was used to construct heatmap. The miRNAs of hub genes were predicted *via* the Mirwalk database (an online tool for predicting miRNA binding sites) [16] Transcriptional Regulatory Relationships Unraveled by Sentence-based Text mining (TRRUST) database, a versatile database for the study of the transcriptional regulation involved in human diseases, was employed to identify TFs regulating the hub genes [17]. The TF-mRNA-miRNA network was visualized in diagrams with the help of the Cytoscape software (Version 3.10.1).

### 2.5. Immune profile analysis

We refined the tumor microenvironment analysis as follows: Firstly, the R package “CIBERSORT” (v0.1.0) was applied to deconvolute the normalized gene expression matrix (log2(FPKM+1)) with 1000 permutations to estimate proportions of 22 immune cell types; secondly, stromal score, immune score, and tumor purity were calculated using the “estimate” package (v1.0.13) with default parameters; further, single-sample gene set enrichment analysis (ssGSEA) for 28 immune signatures was performed via the “GSVA” package (v1.46.0), normalized by z-score; finally, Wilcoxon rank-sum test (*p* < 0.05) assessed immune cell differences between high- and low-risk groups, visualized via the “pheatmap” package [18].

### 2.6. Cell culture and tissue specimens

HPDE6-C7 (human normal pancreatic ductal epithelial cell line) and Capan-1, COLO357, PANC-1, CFPAC-1, MIA PaCa-2 (human pancreatic adenocarcinoma cell line) derived from the Shanghai Cell Bank of the Chinese Academy of Sciences (Shanghai, China) were cultivated in 10% fetal bovine serum (SORFA, China) containing high glucose DMEM (Gibco, USA) containing 10% fetal bovine serum (SORFA, China). The cell culture conditions were 5% CO2, 37°C and humidity.

Tissue samples for RT-qPCR were obtained from the First Affiliated Hospital of Chongqing Medical University, including primary tumors (5 samples) and paired surgical margin tissues as normal tissues (5 samples). Informed written consent for both diagnostic and research purposes was obtained from all patients. The study protocol was approved by the ethics committee of The Third Hospital of Mianyang (Sichuan Mental Health Center) (approval notice: 2022-14-2).

### 2.7. RNA extraction, quantitative real-time PCR analysis and RNA interference

Cell and tissue total RNA was obtained using Trizol reagent (Takara, Japan). RNA was reverse transcribed into cDNA by using PrimeScript RT Kit (Takara, RR037A). RT-qPCR analysis was realized with the application of SYBR Green Premix Pro Taq HS qPCR Kit (Accurate Biology, China). Relative mRNA levels were computed through the 2^-ΔΔct^ algorithm. Primers for PCR were lined up in S1 Table. qPCR primers were designed using Primer Premier 5.0 and validated for specificity by BLAST. Primer sequences were as follows: PDP1 Forward: 5’-CAGGTGCTGGAGAAGGACAT-3’ Reverse: 5’-TGGTGGTGTTGCTGTTGAGT-3’ (Amplicon: 152 bp) LIPT1 Forward: 5’-GCTGGCTACCTGAACCTGAA-3’ Reverse: 5’-CAGCACCTTCCAGGTCTCTT-3’ (Amplicon: 189 bp) Primers were synthesized by Tsingke Biotechnology, dissolved in nuclease-free water (final concentration 10 μM), with amplification efficiency >95%.

siRNA targeting human LIPT1 were procured from Tsingke (Tsingke Biotechnology, China), Capan-1 and CFPAC-1 cell lines were selected for knockdowning and transfected siLIPT1 and siNC for 48 hours. All manipulations followed the user manual, and Lipofectamine 2000 (Invitrogen, USA) was employed for cell transfection. The cells 48 h post transfection were harvested for later detection. WB assays were implemented to testify cells’ knockdowning efficiency, which GAPDH as a loading control. Following the LIPT1-siRNA squences:

LIPT1-SiRNA1: GGAATATTCTCTGTGAAAA

LIPT2-SiRNA2: GAAGCACCTGATCATTGGT

LIPT1-SiRNA3: GGAAAUACGUGACAAAUU

### 2.8. Western blot analysis

Whole-cell lysates were obtained on ice using RIPA buffer that contained 1 mM PMSF and 1 mM NaF (Beyotime Technology, China). The supernatants were collected with 5-min centrifugation at 13,000 g, and total protein concentrations were quantified via the BCA Protein Assay Kit (Beyotime Technology, China). The extracts were added to 5 × loading buffer (Mengbio, China) and denatured by boiling at 100°C for 10 min. Aliquots of 40 μg protein lysates were electrophoresed on 10% sodium dodecyl sulfate-polyacrylamide gel electrophoresis (Epizyme, China) and transferred to PVDF membranes (Invitrogen, USA). The membranes were blocked with blocking buffer (Beyotime Technology, China). Primary antibodies against LIPT1 (Simga, AV48784), GAPDH (Huabio, 12D6) together with cells underwent 4°C overnight incubation. Another 2-h incubation was done with HRP-conjugated secondary antibodies (Servicebio, G1213, China). The HRP Substrate kit (Merckmillipore Corporation, Germany) visualized the blots.

### 2.9. Cell proliferation assays and colony formation assays

The cells seeded in 96-well plates (2000/well) underwent a culture at 5% CO_2_ and 37°C, followed by incubation with CCK-8 reagent (Biosharp, China) at 0, 24, 48 and 72 h, which lasted for 2 h per incubation. A microplate reader (Infinite 2000-PRO, TECAN, Switzerland) was utilized for absorbance (450 nm) detection.

The culture of cells implanted in six-well plates (1000, 2000, and 3000/well) lasted for 10–14 days. Following fixation in 4% paraformaldehyde (Servicebio, China), the CanoScan 8800F MOEL-85 scanner (CanoScan, Japan) was employed to scan and count the surviving colonies, which were then visualized by Gentian violet (Servicebio, China). Three independent replicates were run.

### 2.10. Transwell cell migration and invasion experiments

Transwell chambers (Corning Inc., USA; 6.5-mm diameter inserts, 8-um pore size) were adopted for cell migration and invasion assessment. A Matrigel glue (Becton, Dickinson and Company, USA) was added to the Transwell membrane to perform a cell invasiveness assay. 48 h post inoculation, cells on the lower surface of the chamber were photographed and counted under a phase contrast microscope (Leica, Germany). Each experiment was run independently 3 times.

### 2.11. Statistical methods

Tow statistical methods, R version 4.1.3 and GraphPad Prism v. 8.01 (GraphPad Software, La Jolla, CA, USA), were adopted for data processing. Data satisfying normal distribution were compared by Student’s t test, and non−parametric data were examined *via* the Mann-Whitney U test. The analyses of categorical variables were achieved by Fisher’s exact or Chi-square test. The ANOVA method was utilized for comparing multiple groups of continuous variables. Kaplan-Meier survival curves together with the log-rank test examined OS and DFS. Uni- and multi-variable Cox regression methods were employed for survival analysis. GO, KEGG function enrichment analyses, and GSEA were conducted on the R language software and “clusterProfiler” package. ESTIMATE, ssGSEA, and CIBERSORT algorithms were done using the “estimate,” “GSVA” and “CIBERSORT” packages in R software, separately. Statistical significance was assumed if *p* < 0.05.

## 3. Results

### 3.1. The expression patterns and genetic variations in CRGs in pancreatic cancer

**Fig 1:**
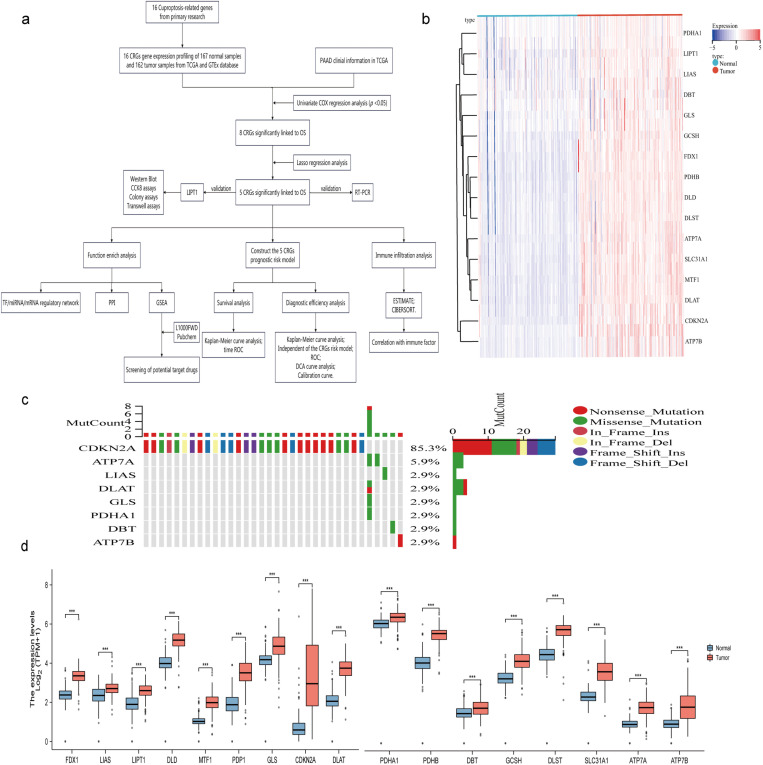
Flow chart and hierarchical clustering analysis of cuproposis-related genes (CRGs). a Flow chart of the overall study design. b Heatmaps of the CRGs. The up-regulated CRGs are marked in red, whereas the down-regulated DEARGs are marked in blue. c The mutation frequency of 17 cuproptosis regulators in 174 samples from TCGA-PAAD. d The expression dristribution of 16 CRGs between normal samples and tumor samples in TCGA-PAAD.

The flow chart of our study design is shown in Fig 1a. The expression of 16 CRGs in 167 normal samples and 162 pancreatic cancer samples is shown in heat map (Fig 1b). Genetic variants plays a crucial role in the origin and progression of cancer. Herein, the frequency of genetic variants in CRGs in pancreatic cancer samples were analyzed. The results suggested that CDKN2A had the highest mutation frequency of 85.3%, while ATP7A (5.9%) was a commonly mutated gene (Fig 1c). Box line plots were generated to compare the expression pattern of CRGs in pancreatic cancer samples and paired non-cancerous samples. It was found that the expression of CRGs in cancerous tissues versus non-cancerous tissues increased obviously (Fig 1d).

### 3.2. A survival prediction model for pancreatic cancer established by CRGs

**Fig 2:**
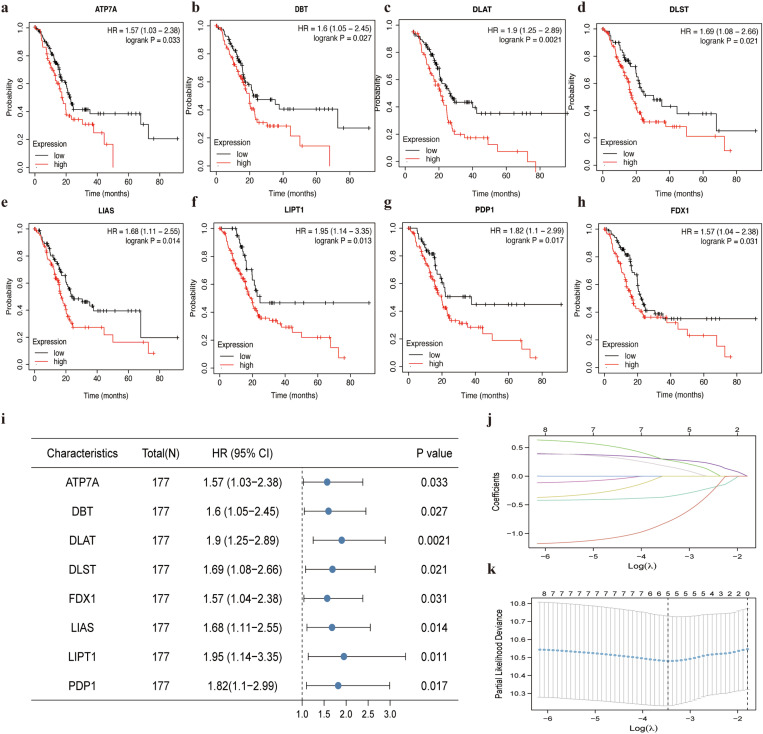
Prediction of clinical correlation of CRGs. a-h Kaplan-Meier analysis of the higher expression and lower expression of candidate Hub genes (p < **0.05). i Forest plot of prognostic differentially expressed candidate Hub genes. j Candidate Hub genes from the univariate Cox regression analysis were ﬁltered by the LASSO algorithm. k LASSO coefﬁcient proﬁles.**

In this study, Kaplan-Meier survival curves were utilized to determine whether CRGs could be used as prognostic biological markers for pancreatic cancer. Notably, only eight CRGs (ATP7A, HR = 1.57, *p* = 0.033; DBT, HR = 1.6, *p* = 0.027; DLAT, HR = 1.9, *p* = 0.0021; DLST, HR = 1.69, *p* = 0.021; LIAS, HR = 1.68, *p* = 0.014; LIPT1, HR = 1.95, *p* = 0.0013; PDP1, HR = 1.82, *p* = 0.017; FDX1, HR = 1.57, *p* = 0.031) expressions are associated with pancreatic cancer prognosis, with patients with high levels of these CRGs having a more unfavorable prognosis (Fig 2a-h). Meanwhile, the forest plot of prognostically differentially expressed candidate hub genes is shown in Fig 2i. Subsequently, the LASSO-Cox model for the survival forecasting in pancreatic cancer patients was constructed with the optimal candidate genes screened from uni-variable Cox regression genes (Fig 2j). Ultimately, 5 candidate gene signatures showing the optimal log λ value were generated according to the LASSO-Cox model (Fig 2k). PDP1, DLAT, DBT, LIAS and LITP1 were eventually chosen as the hub genes. Based on OS data, the regression coefficients were utilized for survival prediction model construction. Next, the expression profiles of five hub genes in gastrointestinal tumors were examined. The results demonstrated that the expression was equally dysregulated in esophageal, gastric, hepatic and colorectal cancer types, with most expressions upregulated compared to normal tissues (S1 Fig. a-d).

### 3.3. Pancreatic cancer survival and diagnosis forecasted by PDP1, DLAT, DBT, LIAS and LITP1 expression profiles

**Fig 3:**
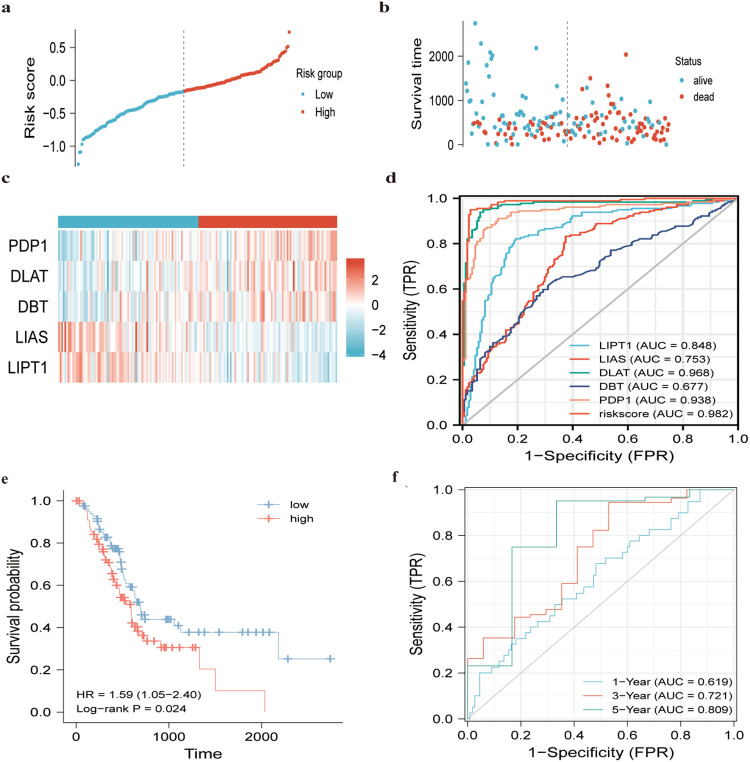
Evaluation of the prognostic model based on hub genes. a Distribution of riskscore of high risk and low risk PAAD patients based on the hub genes-based prognostic model. b The scatter plot showed the correlation between the expression of hub genes in high risk and low risk PAAD patients. c The heatmap showed the correlation between the survival status of high risk and low risk PAAD patients. d AUC values of the riskscore and monogene. e Kaplan-Meier analysis of the high risk and low risk groups of PAAD patients. f ROC curves of the riskscore of PAAD patients at 1, 3, and 5 years. AUC, area under the receiver operating characteristic curve.

Subsequently, risk score distribution and expression patterns of PDP1, DLAT, DBT, LIAS and LITP1 in pancreatic cancer samples were analyzed (Fig 3a-b). The group at higher risk for CRGs had a worse prognosis. The survival statuses of high- and low-risk PAAD patients were further visualized using a heatmap (Fig 3c). ROC analysis was carried out to clarify the effectiveness of this prediction model for the diagnosis of pancreatic cancer.. This prediction model had the highest area under the curve (AUC = 0.982), followed by DLAT (AUC = 0.968), PDP1 (AUC = 0.938, LIPT1 (AUC = 0.848), LIAS (AUC = 0.753), and DBT (AUC = 0.677) (Fig 3d). To avoid false positives and false negatives of the ROC curve, DCA was further performed to compare the diagnostic performance between risk scores and clinicopathological features. In the DCA diagram, the corresponding model with a closer curve to the baseline (all positive and all negative) was linked to a poorer clinical predictive performance. In accordance with the DCA plot, the curves of this model outperformed the curves of other clinicopathological features for diagnosing pancreatic cancer (S1 Fig.). Kaplan-Meier curves suggested that high-risk PAAD patients had a shorter survival time than the low-risk population (Fig 3e). ROC curves were constructed over time to assess the model validity. The AUC of the TCGA-PAAD dataset was 0.619, 0.721 and 0.809 at the time points of 1, 3 and 5 year, respectively (Fig 3f). Fig 3d and S1 Fig demonstrate that the 5-CRGs risk score model achieved the highest clinical net benefit in DCA. For example, at a threshold probability of *p*_t _= 0.3 (i.e., recommending intervention for patients with ≥30% risk), the net benefit of the model was 0.52, significantly outperforming TNM staging (0.32) and age (0.18) (S1 Fig a-c). This suggests that applying the model to guide clinical decisions (e.g., early chemotherapy for high-risk patients) maximizes clinical benefits while reducing overtreatment.

### 3.4. CRGs as independent prognostic parameters for pancreatic cancer

**Fig 4:**
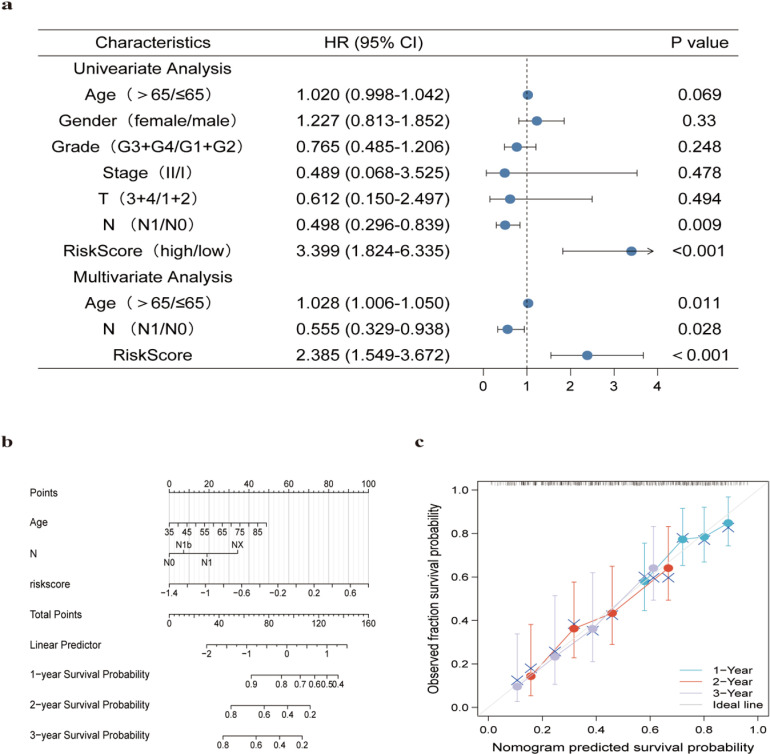
Comparative analysis of diagnostic efﬁciency between the riskscore and clinicopathological characteristics, and construction and evaluation of a prognostic nomogram. a Univariate and multivariate analyses of prognostic factors. (T, N indicate T stage and N stage. The calculation of M stage is ignored due to too little data). b The nomogram predicts the probability of the 1-, 2-, and 3-year OS. c Calibration curves of the CRG-based riskscore in predicting the OS probability at 1/2/3 years.

Age, gender, grade, stage, T stage, N stage, and RiskScore data were extracted from TCGA-PAAD. Uni- and multi-variable Cox regression methods were utilized to check whether these characteristics were correlated with prognosis and to test whether they met independent prognostic indicators. Univariate Cox regression resulted in age, pathological N stage, and RiskScore being associated with prognosis, while multifactorial Cox regression further indicated that age, pathological N stage, and RiskScore were independent prognostic indicators (Fig 4a). Next, to better grasp the significance of the model in tumor development, its prognostic value for clinicopathological stratification was further investigated. Regardless of age ≤ 65 or age > 65, the high-risk population was linked to a poorer prognosis (S2 Fig. a-b). Specifically, the high-risk group was associated with poorer OS in stage T1 + 2 (HR = 1.57, *p* = 0.049) and stage T3 + 4 (HR = 1.57, *p* = 0.049) of pancreatic cancer patients (S2 Fig. c-d). Furthermore, it was noticed that OS at stage M0 (HR = 9.33, *p* ＜ 0.001), M1 + x (HR = 2.27, *p* = 0.006), N0 (HR = 12.08, *p* ＜ 0.001), and N1 + x (HR = 3.28, *p* ＜ 0.001) were related to high-risk group, in which pancreatic cancer patients had a shorter survival time at the same time (S2 Fig. e-h). Then, a column line graph with prognostic factors containing age, pathological N stage, and RiskScore was constructed. This reveals a C index as high as 0.673. Therefore, the model may have the potential of accurate prognostic prediction for pancreatic cancer (Fig 4b). To assess the robustness of the prediction results, calibration curves for the 1-, 2-, and 3-year OS probability were predicted based on risk scores for CRGs. The deviation scale was noted to be acceptable, and the predicted curves for the 1-, 2-, and 3-year OS were relatively close to the ideal dashed line (Fig 4c).

### 3.5. The associated mechanism of hub genes

**Fig 5:**
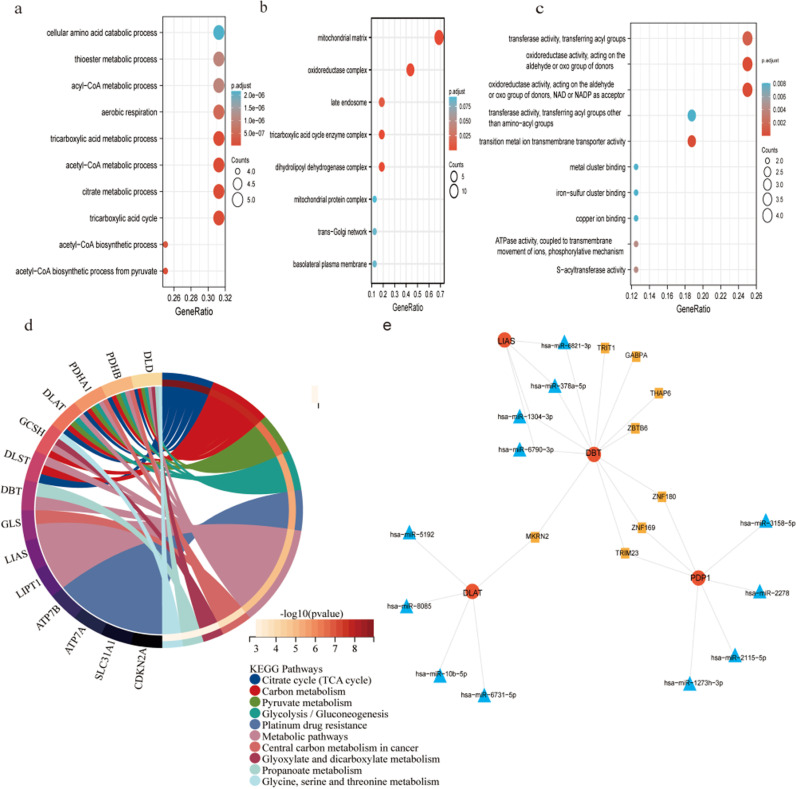
Exploring the mechanism of action of hub genes. a-d Functional enrichment(GO and KEGG) of 5 hub genes. e View of the transcription factor (TF/mRNA/miRNA regulatory network. (TFs, mRNAs and miRNAs are represented by pale brown squares, red circles and deep blue triangles, respectively.).

To clarify the mechanism of the hub genes (PDP1, DLAT, DBT, LIAS and LITP1), functional enrichment analysis was firstly performed.. Five hub genes were identified to participate in numerous biological processes. The top three enriched Biological processes (BP) include TCA cycle, citrate metabolic process and acetyl-CoA metabolic process (Fig 5a). The top three enriched Cellular components (CC) consist of mitochondrial matrix, oxidoreductase complex and dihydrolipoyl dehydrogenase complex (Fig 5b). Molecular function (MF) is mainly involved in oxidoreductase activity, acting on the aldehyde or oxo group of donors and NAD or NADP as acceptor (Fig 5c). oOn the other hand, KEGG was significantly enriched mainly in Citrate cycle (TCA cycle), Carbon metabolism and Pyruvate metabolism (Fig 5d). A hub genes-associated TF/miRNA/mRNA network was developed by analyzing the relations between TFs, mRNAs, and miRNAs of 5 CRGs. On the ground of the miRNA-DECRG forecast data on the Starbase tool and TF-DECRG predictions from the chEA3, a network comprising eight TFs, twelve miRNAs, and four targeted mRNAs was found (Fig 5e).

### 3.6. CRGs are closely relevant to the TME in pancreatic cancer

**Fig 6:**
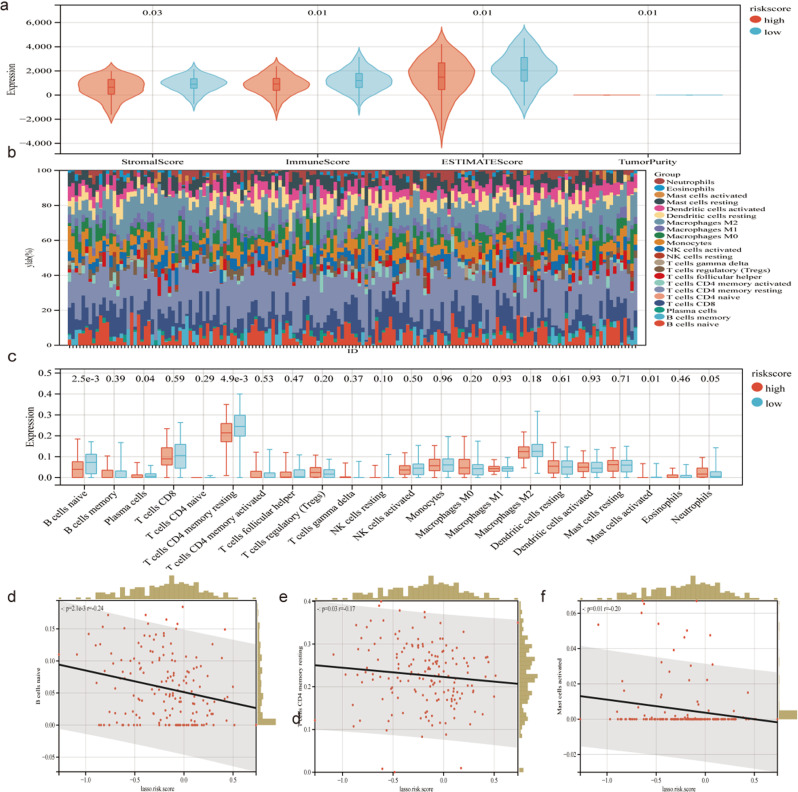
The immune phenotype landscape in PAAD and normal and co-expression patterns of hub genes and immune cell subpopulations. a The stromal score, Immune score, ESTIMATE score and Tumor purity. b CIBERSORT algorithm revealed the abundance of different immune cells in normal pancreas tissues and PAAD tissues c Bar plot showing the relative proportions of 22 immune cell populations in TCGA-PAAD. d-f Pearson correlation analysis of different infiltrating immune cell subpopulations.

With the TCGA-PAAD cohort-derived clinical data, the role of CRGs in the immune infiltration of pancreatic cancer was quantitatively elucidated using the ESTIMATE algorithm. It was found that stromal, immune, and ESTIMATE score increased while the tumor purity in the low-scoring group decreased (Fig 6a). Additionally, the abundance of different immune cells in normal pancreatic tissue and pancreatic cancer tissue was computed by the CIBERSORT algorithm. There are diverse immune cell types involved in this process (Fig 6b). The relative percentages of the 22 immune cell populations in TCGA-PAAD is shown in Bar plot, and the low-scoring groups had more B cells native, T cells CD4 memory resting, Mast cells activated than the high-scoring groups (Fig 6c). Meanwhile, Pearson correlation analysis showed that natural B-cells, T-cell CD4 memory resting, and mast cell activation were negatively and statistically significantly correlated with the LASSO risk score (Fig 6d-f).

### 3.7. Expression characteristics of five Hub Genes in Pancreatic Cancer Cell Lines and Tissue Specimens

**Fig 7:**
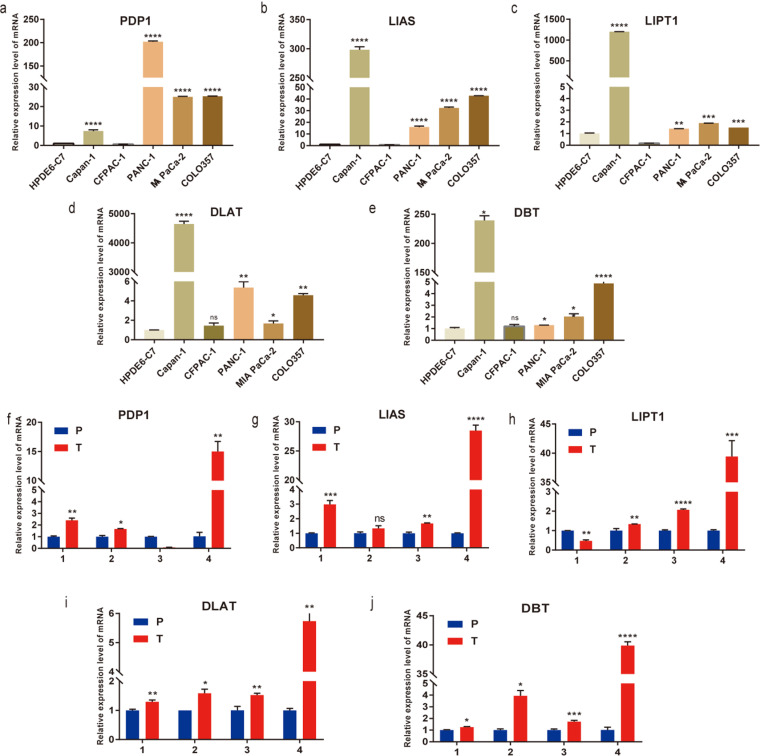
mRNA expression of hub genes. a-e PDP1, LIAS, LIPT1, DLAT, DBT mRNA expression in five PC cell lines. f-j PDP1, LIAS, LIPT1, DLAT, DBT mRNA expression in five PC Paired tissues.

To clarify the presence of CRGs dysregulation in pancreatic cancer samples, expression characteristics of five genes were further validated by RT-qPCR. PDP1, DLAT, DBT, LIAS and LITP1 were determined to be markedly higher at mRNA levels in pancreatic cancer Capan-1, CFPAC-1, PANC-1, MIA PaCa-2, and COLO357 cells than in normal HPDE6-C7 (Fig 7a–e). Similarly, their expression was obviously higher in paired pancreatic cancer tissues, compared to paracancerous tissues (Fig 7f–j). These verification results were all consistent with our primary screening and identification of Five DECRGs.

### 3.8. LIPT1 knockdown inhibits proliferation, migration and isinvasion in pancreatic cell lines

**Fig 8:**
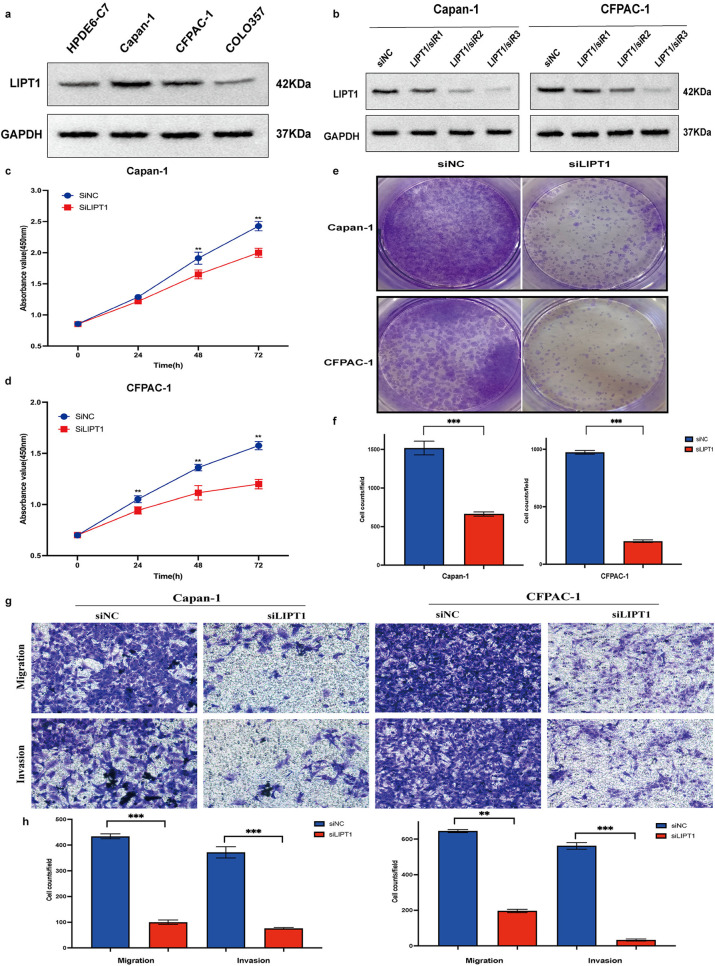
LIPT1 promotes proliferation, invasion and migration in pancreatic cancer cells. a The protein expression levels of LIPT1 in normal pancreatic cell line and five pancreatic cancer cell lines. b The three independent siRNAs knockdown efciency of LIPT1 in Capan-1 and CFPAC-1 cell lines by western blot. c-d Measurement of cell proliferation of the Capan-1 and CFPAC-1 cells transfected with the control siRNA or the siLIPT1 by CCK8. e-f Representative images and quantitative analyses of colony formation assay in the Capan-1 and CFPAC-1 cells transfected with the control siRNA or the siLIPT1. g-h Representative images and quantitative analyses of transwell assay in the Capan-1 and CFPAC-1 cells transfected with the control siRNA or the siLIPT1 (*p < 0.05, **p < 0.01, ***p < 0.001).

In privous multivariate Cox regression analysis, LIPT1 showed a higher risk coefficient (HR = 1.95, *p* = 0.0013) indicating its stronger independent predictive value for patient outcomes. RT-qPCR results revealed that LIPT1 exhibited markedly higher mRNA expression levels in pancreatic cancer cell lines (e.g., Capan-1, CFPAC-1) and clinical tissues compared to normal pancreatic cells (HPDE6-C7) and normal pancreatic tissues, suggesting its potential pro-oncogenic activity in pancreatic cancer. It was selected for further biological function validation in pancreatic cancer cells. Firstly, the protein expression of LIPT1 in pancreatic cancer cell lines was teste. WB indicated that the expression of LIPT1 were significantly elevated in pancreatic cancer cell lines (Capan-1, CFPAC-1 and COLO357) compared to normal pancreatic cells (HPDE6-C7) (Fig 8a). Afterwards, LIPT1 knockdowning cell lines (siLIPT1) in Capan-1 and CFPAC-1 cell lines were successfully transfected (Fig 8b). CCK-8 and colony formation assays elucidated that LIPT1 knock downing markedly restricted the proliferation of pancreatic cancer cell lines (Fig 8c-f). Further, more attention was paid to the possible impacts of LIPT1 on metastasis. There were notably fewer LIPT1 knockdowning cells passing through the transwell chamber in Capan-1 and CFPAC-1 cell lines. Furthermore, LIPT1-deficient panceratic cancer cells invading the Matrigel barrier were remarkably decreased as compared to control cells (Fig 8g-h). These results indicated that LIPT1 might promote pancreatic cancer cell proliferation, migration and invasion in vitro. LIPT1 is likely to be a potential target for hepatocellular carcinoma therapy.

## 4. Discussion

In this study, 16 CRGs were collected from existing literature. Then, the RNA levels of CRGs between neoplastic and adjacent non-neoplastic tissues in the TCGA and GTEx cohorts were calculated. Hence, the expression of 16 CRGs was noticeably higher in pancreatic cancer than in non-neoplastic tissues, and 8 of these genes were associated with pancreatic cancer prognosis. Following that, a LASSO-Cox model was constructed for survival prognostication by incorporating the uni-variable Cox regression genes. Five essential CRGs, PDP1, DLAT, DBT, LIAS and LIPT1, were finally selected for prognostic signature construction. The higher AUCs of this prognostic signature shown by the ROC curves suggest advanced predictive performance. Moreover, Kaplan–Meier calculations indicated that OS was significantly prolonged in low-risk individuals compared to high-risk individuals. A conventional nomogram was also introduced and reflected the prognostic accuracy of model for PAAD. It is worth noting that these conclusions were derived from routine assessments (i.e., AUC values in time-dependent ROC analyses), as well as from the DCA results. This stems from a fact that we understand the need to maximize the merit of tolerating false positives and false negatives in situations that are unavoidable in clinical practice.

These five genes perform significant roles in the development and metastasis of diverse tumors. PDP1 overexpression has been reported to be present in human prostate and pancreatic cancers, promoting cell proliferation and tumor growth [19,20]. DLAT crucially engages in glucose metabolism and the TCA cycle. Goh et al. reported an up-regulation of DLAT in gastric cancer cells and they illustrated that siRNA-mediated knockdown of DLAT increased pyruvate levels [8,21]. Furthermore, DLAT can be an independent risk prognostic parameter for OS and RFS in patients with hepatocellular carcinoma [22]. Overexpression of dihydrolipoamide branched chain transacylase E2 (DBT), which encodes one of the three subunits of BCKD complex, inhibits the invasiveness of renal clear cell carcinoma (KIRC). This shows a relationship with immune infiltration [23]. Lipoic acid synthetase (LIAS) engages in the synthetic process of mitochondria-related metabolic enzymes, showing roles in the regulation of energy metabolism and oxidative stress [24]. LIAS mutations impairs mitochondrial energy metabolism [25] Moreover, high LIAS expression has been documented to be related to a favorable prognosis in patients with KIRC, rectal adenocarcinoma (READ), breast carcinoma, and ovarian carcinoma [26]. LIPT1, encoding fatty acyltransferase 1, is a crucial regulator of lipoic acid (LA) transport, while LA significantly participates in the TCA cycle, as well as mitochondrial metabolism in tumor cells [27–29]. LIPT1 has been verified to strengthen the proliferating, invading, and migrating behaviors of LIHC cells, while LIPT1 is related to a favorable prognosis of individuals suffering from urothelial cancer or melanoma [30–32]. Overall, these findings supported the notion that CRGs may influence the progression and prognosis of PAAD, thereby illustrating the predictive performance of the five-CRGs signature model.

Furthermore, CRGs are enriched in pathways related to the TCA cycle, citric acid metabolic processes, pyruvate metabolism, glycolysis, and central carbon metabolism in cancer. TCA cycle is a key mechanism involving in energy metabolism, macromolecular synthesis, and redox homeostasis. Studies have demonstrated the associations between abnormalities of the TCA cycle and various cancers [33–35]. Of note, various oncogenes and anti-oncogenes mediate the uptake and catabolism of fuel sources in the TCA cycle *via* modulating fuel transporter protein expression and/or cycling enzyme activity in cancerous cells [36]. Aerobic glycolysis, also called the Warburg effect, is a characteristic metabolic mechanism usually develops in cancerous cells. Many tumor cells are partly determined by a metabolic switch known as the Warburg effect, where glycolytic carbon flux is highly elevated and oxidative phosphorylation is remarkably attenuated [37,38] Multiple types of tumors restrain the pyruvate oxidation to highly proliferating tumor cells [39]. Tumorigenesis relies on cell metabolism reprogramming directly and indirectly resulting from oncogenic mutations. Changes in intracellular and extracellular metabolites following cancer-related metabolic reprogramming exert far-reaching have impacts on gene expression, cell differentiation, and TME. The data presented herein suggest that the effect of cellular copper levels on metabolism-related pathways may be a regulatory mechanism for copper toxicity in cancer.

TME is a heterogeneous and complicated population comprising tumor, stroma, and endothelial cells, in which immune cells exhibit vital modulatory functions in tumor growth. There is evidence that B cells perform an anti-cancer role through various mechanisms. For instance, they enhance the activity of cytotoxic T cells and activate antibody-dependent cytotoxicity [40,41]. CD4 T cells can target tumor cells in a number of ways, either by direct elimination of tumor cells through cytolytic mechanisms or by indirect regulation of TME [42]. In TME, MCs have antitumor properties. Once activated and degranulated, these cells are transformed to be highly pro-inflammatory and actively recruited in the innate immune system to orchestrate anti-tumor immune responses [43]. In current study, there are more naive B cells, T cells CD4 memory resting and Mast cells activated in the low-scoring population than the high-scoring population, suggesting a better immune reaction in these low-scoring individuals. Additionally, relatively higher stromal, immune, and ESTIMATE scores were detected in the low-scoring individuals. These findings suggest that the potential of CRGs as a categorization of populations with high and low CRGs plays a crucial role in differentiating TME status in PAAD.

The mRNA expression of these 5 hub genes were experimentally validated using RT-qPCR experiments in 5 pancreatic cancer cell lines and pancreatic cancer paired tissues, respectively. Then Capan-1 and CFPAC-1 pancreatic cancer cell lines were selected for further experiments on LIPT1. The LIPT1 protein delivers a lipid-based fragment of lipoyl lipoate to glycine cleavage system protein H (GCSH) and the E2 subunit of 2-oxoacid dehydrogenase, which is involved in the metabolism of lipoic acid [25,44]. Mutations in the LIPT1 gene can cause a few hereditary diseases [45]. The results in previous studies on the role of LIPT1 in tumors are inconsistent. Some studies have shown that LIPT1 plays an oncogenic role in patients with uroepithelial carcinoma or melanoma [31,32], but others have found that LIPT1 expression is up-regulated in LIHC, which is an independent prognostic factor for the poor prognosis of LIHC [30]. This suggests that the gene plays different roles in different types of tumors, which is a situation that exists [46]. The experimental validation of this study for the biological function of LIPT1 suggests that LIPT1 promotes the proliferation, migration and invasion of pancreatic cancer, supporting the view that LIPT1 promotes the development of tumorigenesis, but the specific regulatory mechanism needs to be further investigated. In addition, their molecular mechanism involving in tumor metastasis and immunity in this malignancy require in-depth explorations. Second, the sample size of this trial was small, and the clinical data were primarily from the TCGA cohort, most of whom were white North American patients. Immune cell infiltration assessed only based on the algorithm also awaits future studies. This study has the following limitations, which will be addressed in future optimizations: Firstly, the clinical data were primarily sourced from the TCGA database and predominantly involve North American Caucasian populations, and the lack of data from Asian or other ethnic groups may limit the model’s generalizability. Additionally, RT-qPCR validation was performed on only 5 pairs of tumor and adjacent tissues, necessitating an expanded sample size. Secondly, the analysis of immune infiltration in the tumor microenvironment relied on algorithms such as CIBERSORT, and further experimental validation using flow cytometry or immunohistochemistry is required. Furthermore, although the pro-tumorigenic role of LIPT1 was validated, its specific regulatory mechanisms in cuproptosis—such as through the TCA cycle or lipoylated protein pathways—remain unelucidated, and follow-up studies will employ CRISPR/Cas9 gene editing, metabolomics, and PDX mouse models to explore these mechanisms. Lastly, the current prognostic model was constructed solely based on transcriptomic data; integrating imaging data, pathological grading, and validation through multicenter cohorts is essential to enhance its clinical applicability. Future research will refine the study design to explore the translational potential of cuproptosis-related genes (CRGs) in pancreatic cancer diagnosis and treatment.

## 5. Conclusion

Altogether, five CRGs relevant to pancreatic cancer prognosis were identified. According to RT-qPCR, it can be concluded that the expression of 5 genes is consistent with previous results. LIPT1 may promote proliferation, invasion and migration of pancreatic cancer cell lines. Futhermore, a newly-established and accurate five CRGs-based prognostic model was developed for this malignancy. This work can help to propose novel insights and guide clinical treatment strategies for pancreatic cancer.

## Supporting information

S1 Table List of primers used in real-time PCR. (PDF)

S1 Fig Comparative analysis of diagnostic efﬁciency between the riskscore and clinicopathological characteristics. a-c DCA of the risk score and clinicopathological characteristics at 1/2/3/5 year. (PDF)

S2 Fig Prognostic value in clinicopathological stratification. a&e Prognostic value of the prognostic model based on hub genes in age. b&f Prognostic value of the prognostic model based on hub genes in T stage. c&g Prognostic value of the prognostic model based on hub genes in M stage. d&h Prognostic value of the prognostic model based on hub genes in N stage. (PDF)
