# Supplementary material for: Comprehensive analysis and experiment validation of five cuproptosis-related genes in prognosis, immune infiltration and metabolic characterization of pancreatic cancer
Source: PLoS One. 2025 May 14;20(5):e0323458. doi: 10.1371/journal.pone.0323458 (PMC12077771; doi:10.1371/journal.pone.0323458)
Supplement: S1 Table — (PDF) [file pone.0323458.s001.pdf]

S1 Table. List of primers used in real-time PCR

Primer	Sequence (5′-3′)	Molecular Weight (kDa)	Annealing tem perature (°C)
PDP1-F	GAGGCCAAGAGTGTCGTGAA	6230.88	60
PDP1-R	CGGTGGCCTTAATCGGTGGT	6154.84	
LIAS-F	AGTATGTGAGGAAGCTCGATGTC	7014.47	60
LIAS-R	GGAGGAGGATTTCTTGCAGTCTTA	7438.67	
LIPT1-F	AAACATGCACACGACCGTAATG	6721.33	60
LIPT1-R	GGGACCTGGCAGTTACAAAGTAA	7121.5	
DLAT-F	ACCAAAGCAAGAGAGGGTAAACT	7123.55	60
DLAT-R	AGACATCATGCTAGCCACATCAA	6985.53	
DBT-F	CTCCTGCAGTGTCTCATGATGAA	7014.47	60
DBT-R	ACTTTGGGTGAAGGAGGCAATAT	7167.51	
GAPDH-F	CACTCCTCCACCTTTGACGC	5948.9	60
GAPDH-R	CTGTTGCTGTAGCCAAATTCGT	6716.4	
