# Supplementary material for: Comprehensive analysis and experiment validation of five cuproptosis-related genes in prognosis, immune infiltration and metabolic characterization of pancreatic cancer
Source: PLoS One. 2025 May 14;20(5):e0323458. doi: 10.1371/journal.pone.0323458 (PMC12077771; doi:10.1371/journal.pone.0323458)
Supplement: S1 Fig — (PDF) [file pone.0323458.s002.pdf]

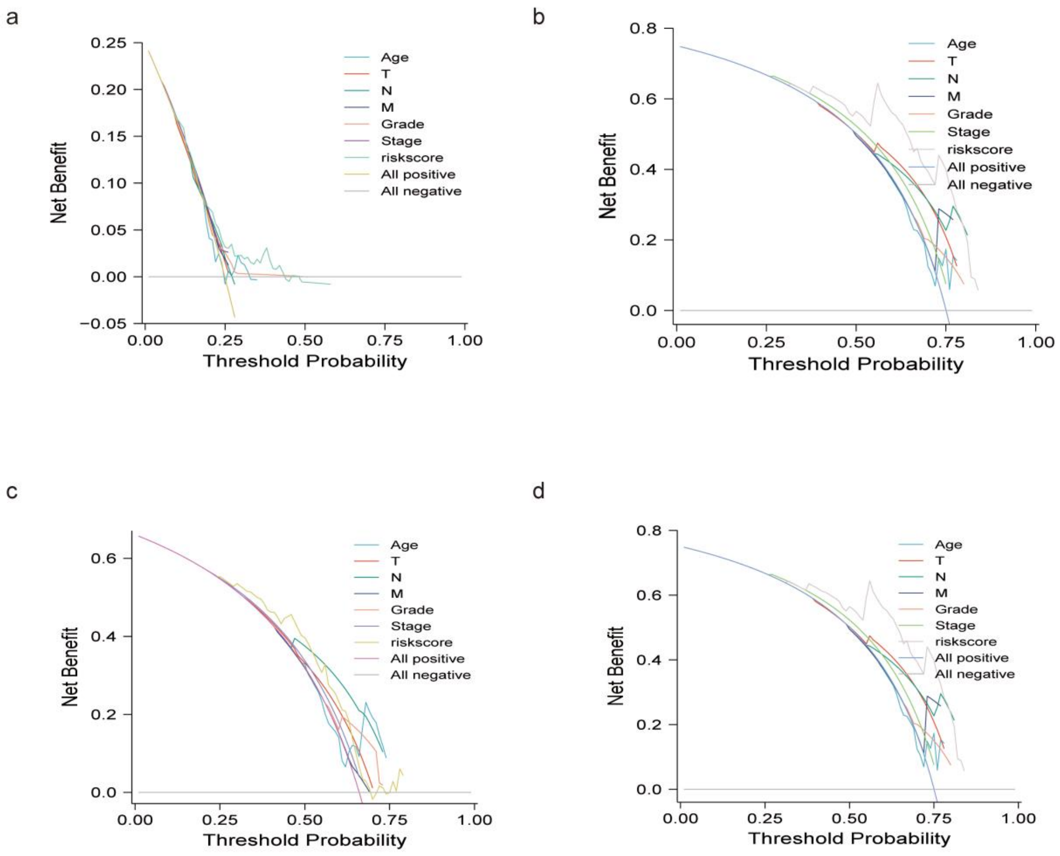

S1 Fig. Comparative analysis of diagnostic efficiency between the riskscore and clinicopathological characteristics. a-c DCA of the risk score and clinicopathological characteristics at 1/2/3/5 year.
