# Supplementary material for: Comprehensive analysis and experiment validation of five cuproptosis-related genes in prognosis, immune infiltration and metabolic characterization of pancreatic cancer
Source: PLoS One. 2025 May 14;20(5):e0323458. doi: 10.1371/journal.pone.0323458 (PMC12077771; doi:10.1371/journal.pone.0323458)
Supplement: S2 Fig — (PDF) [file pone.0323458.s003.pdf]

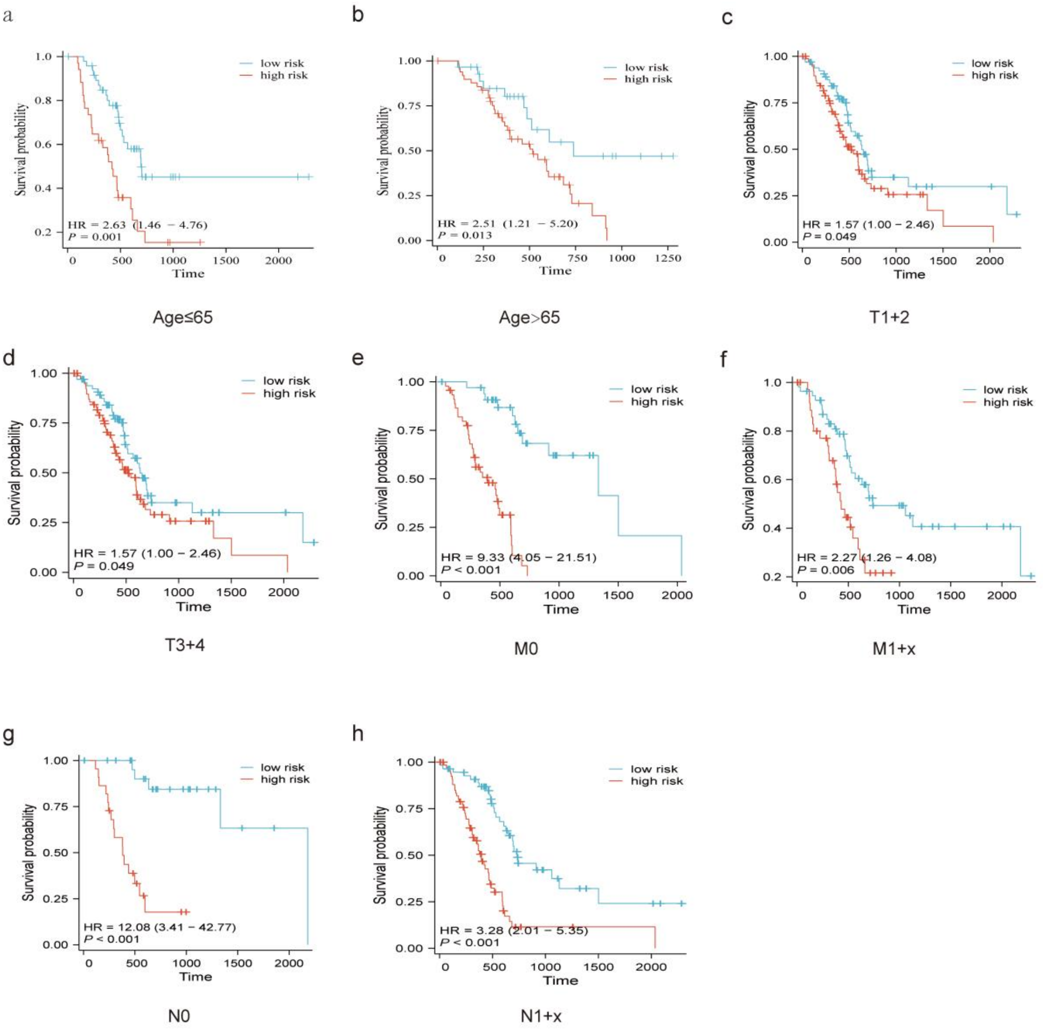

S2 Fig. Prognostic value in clinicopathological stratification. a & e Prognostic value of the prognostic model based on hub genes in age. b & f Prognostic value of the prognostic model based on hub genes in T stage. c & g Prognostic value of the prognostic model based on hub genes in M stage. d & h Prognostic value of the prognostic model based on hub genes in N stage.
